# Guidewires and biopsy forceps: a novel approach for appendiceal orifice closure in endoscopic retrograde appendicitis therapy

**DOI:** 10.1055/a-2869-6072

**Published:** 2026-05-22

**Authors:** Fengtian Yang, Hua Huang, Zhi Wei

**Affiliations:** 1 Department of Gastroenterology 611300 Shandong Second Provincial General HospitalJinanChina

**Fig. 1:**
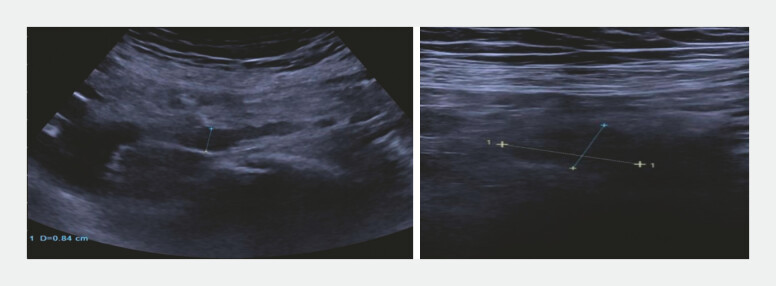
The abdominal ultrasound showed a strip hypoechoic area in the right lower abdomen and enlarged para-appendiceal lymph nodes.

A 72-year-old woman presented with lower abdominal pain that had persisted for 10 days, and exhibited tenderness at McBurneyʼs point. Laboratory investigations revealed a white blood cell count of 11.25 × 10 ^9^ /L, and the abdominal ultrasound showed a strip hypoechoic area in the right lower abdomen and enlarged para-appendiceal lymph nodes ( Fig. 1 ). So, she was referred to our hospital for endoscopic retrograde appendicitis therapy (ERAT).

**Fig. 2:**
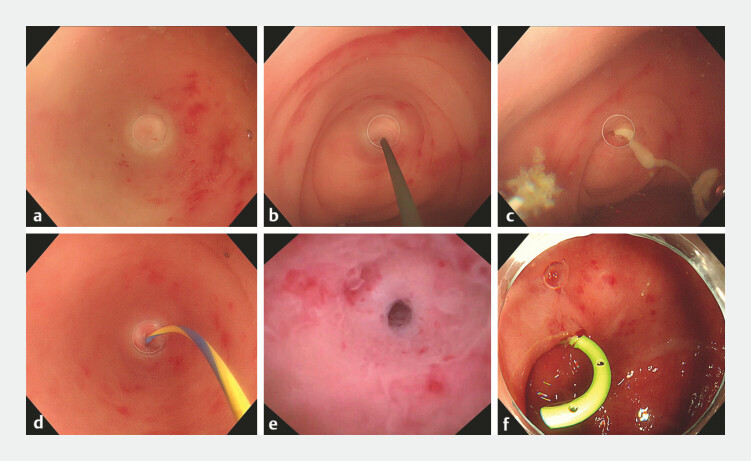
**a** The closure of the appendix was observed. **b** A guidewire was used to explore the direction of appendix. **c** Pus could be seen flowing out of the appendix cavity. **d** A biopsy forceps was employed for blunt dilation of the appendiceal orifice. **e** The mucosa of the appendix cavity was congested and swollen. **f** Stent implantation following endoscopic appendiceal lavage.

ERAT was performed under intravenous anesthesia after informed consent was obtained ( Video 1 ). The colonoscope was inserted into the cecum, and the Gerlach’s valve was pushed aside using the transparent cap; then, the closure of the appendix was observed ( Fig. 2 **a** ).Since the appendiceal orifice could not be identified and the use of a contrast catheter was deemed risky due to the potential for false lumen formation, the direction of the appendix was explored with a guidewire ( Fig. 2 **b** ), pus could be seen flowing out of the appendix cavity ( Fig. 2 **c** ), and a biopsy forceps was used to blunt dilate the appendiceal orifice. While the patient strongly refused the use of X-rays, to ensure that the guidewire entered the true appendiceal lumen rather than a false one, we used the wire to guide the digital cholangioscope into the appendix cavity ( Fig. 2 **d** ), and the mucosa of the appendix cavity was congested and swollen ( Fig. 2 **e** ). During the surgery, the use of a conical cap restricted the directional control of the digital cholangioscope; therefore, a transparent cap was used on the scope tip. Stent implantation following endoscopic appendiceal lavage due to the bottom of lumen stenosis of the appendiceal ( Fig. 2 **f** ).The patientʼs symptoms and signs were relieved after the therapy within 48 hours. Laboratory investigations revealed a white blood cell count of 6.22 × 10 ^9^ /L. Follow up was conducted 6 months later, and the abdominal pain did not recur.

Endoscopic retrograde appendicitis therapy for the management of appendiceal orifice atresia via a guidewire as a surgical exploration tool.Video 1

While case reports have described utilizing a stent retriever for the management of appendiceal orifice stenosis 1 , no feasible endoscopic treatment exists for appendiceal orifice occlusion. In cases where the orifice cannot be identified, the judicious use of guidewires and biopsy forceps may render ERAT feasible.

Endoscopy_UCTN_Code_TTT_1AQ_2AF
